# Performance of the J-CTO score versus other risk scores for predicting procedural difficulty in coronary chronic total occlusion interventions

**DOI:** 10.1080/07853890.2022.2141466

**Published:** 2022-11-02

**Authors:** Wenjie Zuo, Jie Lin, Renhua Sun, Yamin Su, Genshan Ma

**Affiliations:** a Department of Cardiology, Zhongda Hospital, School of Medicine, Southeast University, Nanjing, China; b Department of Cardiology, Taizhou People’s Hospital, Taizhou, China; c Department of Cardiology, The First People’s Hospital of Yancheng City, Yancheng, China; d Department of Cardiology, Affiliated Hospital of Nantong University, Nantong, China

**Keywords:** Risk prediction, chronic total occlusion, percutaneous coronary intervention, J-CTO

## Abstract

**Background:**

Although the Japanese chronic total occlusion (J-CTO) score is widely used to assess the complexity of revascularization for CTO lesions, ambiguous and conflicting results are reported in validation studies. Therefore, we aimed to quantitatively evaluate the effectiveness of the J-CTO score and explore the heterogeneity of its comparison with other CTO scores.

**Methods:**

PubMed, Embase, the Cochrane Library, and ClinicalTrials.gov databases were systematically searched from January 1st, 2011 to December 23rd, 2021. Studies that examined the accuracy of the J-CTO score were eligible. Where feasible, estimates of discrimination and calibration were pooled with a random-effects model. The Prediction model Risk Of Bias ASsessment Tool (PROBAST) was used for risk-of-bias assessment. This study was reported according to PRISMA guidelines and prospectively registered with PROSPERO (CRD42019126161).

**Results:**

Of 28 included studies (*N* = 34,944 lesions), 24 were eligible for meta-analysis. The J-CTO score demonstrated significant discrimination for 30-min wire crossing (summary C-statistic 0.76; 95% CI 0.68–0.84) and technical success (0.68; 95% CI 0.61–0.74) despite significant heterogeneity. Only 19 (33%) of the 58 pairwise comparisons with 14 competing scores that were based on discrimination reported a statistical result. The J-CTO score performed worse (relative difference of C-statistics >5%) in eight out of 33 independent comparisons but better in another 13. Methodological shortcomings resulted from only one study evaluating model calibration appropriately.

**Conclusion:**

The discrimination power of the J-CTO score was useful for time-efficient wire crossing and moderate for angiographic success. Head-to-head comparisons of CTO scores would benefit from standardized reporting and appropriate statistical methods.

**Key messages**
- The J-CTO score has useful discrimination in predicting 30-min wire crossing while performing moderately for technical success.
- After excluding optimism bias, there is insufficient independent evidence supporting the superiority of newly introduced models over the J-CTO score.
- Standardized methodology and assessment are needed to achieve a better understanding of CTO scores, especially for their calibration.

## Introduction

Patients with coronary artery disease often have chronic total occlusions (CTOs), which are defined as complete coronary artery obstructions that persist for at least three months exhibiting thrombolysis in myocardial infarction flow grade zero [1]. This lesion subset is usually more difficult to be treated with percutaneous coronary intervention (PCI) than non-occlusive diseases [2]. Despite significant advances in dedicated devices and recanalizing techniques [3], CTO PCI remains a major challenge for many interventional cardiologists, particularly when they are less experienced. A comprehensive evaluation of patients and their CTO lesions is necessary to achieving success in CTO PCI [4]. For this reason, several scoring systems have been developed to predict procedural complexity and the probability of final success, which can help make clinical decisions, facilitate case selection, and even reduce complications [5].

**Figure 1.**
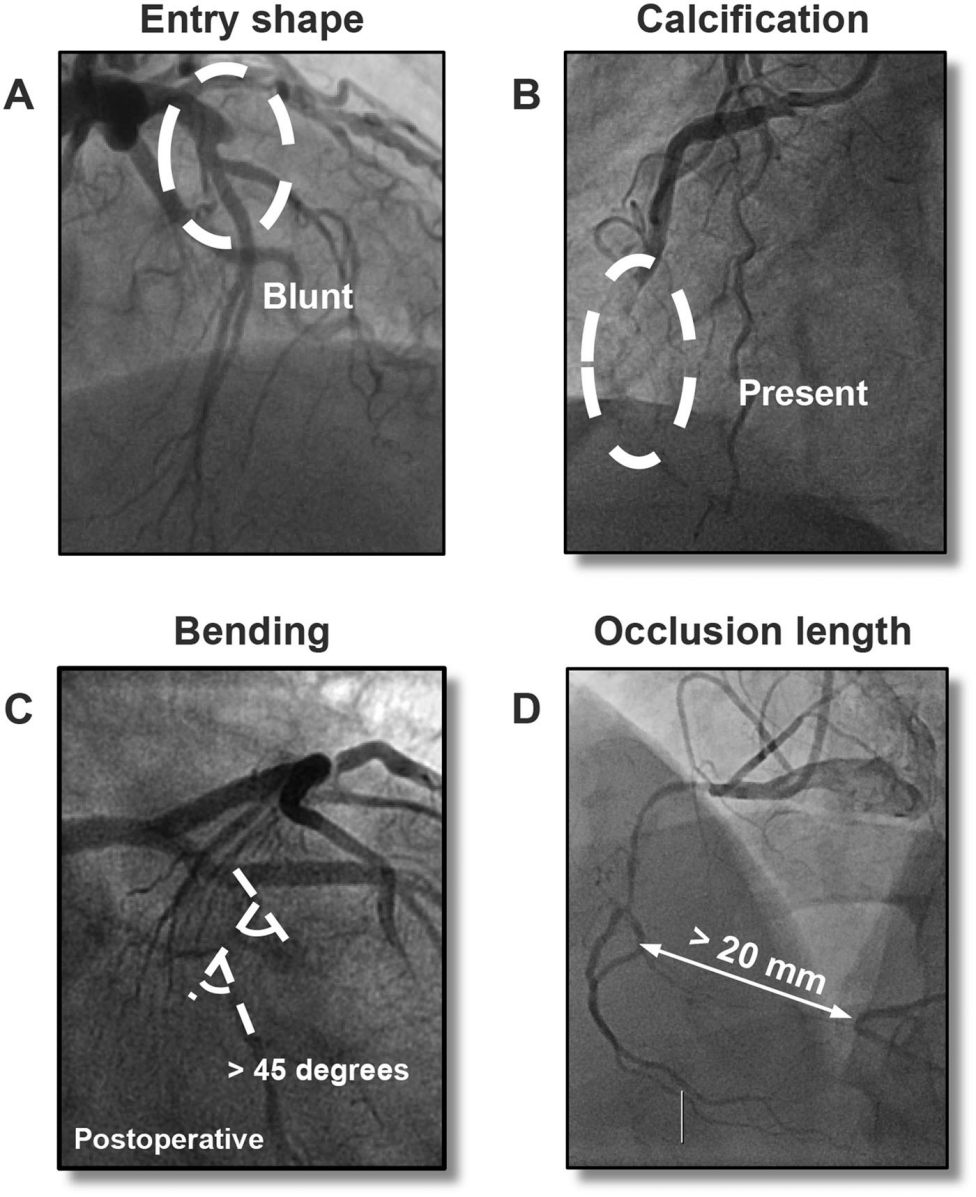
Representative images showing the four morphological characteristics of J-CTO score.

Currently, the most widely used score is the multicentre CTO registry in Japan (J-CTO) score [6,7]. It is composed of five independent variables: blunt stump, calcification, bending >45°, occlusion length ≥20mm, and previously failed attempt (Figure 1). However, its generalizability has not been established due to a relatively low proportion of patients treated by retrograde approach in the original study and inconsistent performance in subsequent studies [8–10]. Moreover, there remains uncertainty about the comparability of the J-CTO and more recent CTO scores. The purpose of this meta-analysis was therefore to (1) provide an overview of evidence on the J-CTO score and its comparators, (2) synthesize its performance for predicting 30-min wire crossing and technical success, and (3) evaluate the methodological quality of validation studies.

## Methods

This meta-analysis was conducted according to the Preferred Reporting Items for Systemic reviews and Meta-Analyses (PRISMA) 2020 checklist [11] and a recent guide by Debray and colleagues [12]. This study was prospectively registered with the International Prospective Register of Systematic Reviews (PROSPERO; CRD42019126161).

### Search strategy and study selection

First, we searched relevant records systematically in PubMed, EMBASE, Cochrane Library, and ClinicalTrials.gov from January 1st, 2011 (because the J-CTO score was first described in 2011) through December 23rd, 2021, using a combination of MeSH and entry term of “CTO” and ‘prediction models’ without any language restriction. Then, duplicate records were automatically removed by a citation manager and the results were checked manually. Lastly, the remaining entries were screened based on titles and abstracts to establish a preliminary list of potentially eligible trials. The search strategy is detailed in Supplementary file.

Studies were included if they compared the performance between the J-CTO and any other scores or only validated the J-CTO score (Table 1). Outcomes of interest were successful 30-min wire crossing and technical success. The exclusion criteria were: (1) neither discrimination nor calibration were reported; (2) non-original articles (e.g. reviews, editorials, and letters); and (3) non-related outcomes. Two independent investigators (Drs Zuo and Lin) were involved in this process and any disagreement was resolved *via* discussion.

**Table 1.** Review question formulated according to the PICOTS system.

Item	Definition
Population	Patients undergoing coronary CTO recanalization
Intervention	Predictive performance of the J-CTO score
Comparator	Other prediction models competing with the J-CTO score
Outcome	Successful guidewire crossing within 30 min Technical success
Timing	Not applicable
Setting	To determine the complexity and difficulty of recanalizing CTO lesions, thus guiding decision-making and procedural planning

CTO: chronic total occlusion; J-CTO: Multicenter CTO Registry of Japan; PICOTS: population, intervention, comparator, outcome, timing, and setting.

### Data extraction and quality assessment

The following items were independently extracted from each study by two reviewers (Drs Sun and Su): data source, time intervals, countries of origin, sample size, number of events, demographics, variables used in models, and metrics of performance. After the extraction process, the results were checked by a senior investigator (Dr. Ma). The overall performance of a model consisted of discrimination and calibration [13]. The discrimination was measured by the C-statistic and a value of >0.75 suggested strong ability [14]. The C-statistics between the J-CTO and other scores was compared, mainly by examining whether their relative gap exceeded 5% or whether there was a statistically significant difference [15]. Net reclassification improvement and integrated discrimination improvement were also recorded. The calibration (the concordance between estimated and observed probabilities) was manifested by Hosmer-Lemeshow statistics (a *P*-value >0.05 indicates good fit) or the total O:E ratio (observed/expected events). If there was missing data, an attempt was made to contact with the corresponding author.

The methodological quality of included studies was assessed using the Prediction model Risk Of Bias ASsessment Tool (PROBAST) [16] across four domains: participants, predictors, outcome, and analysis. Studies must have low risk in all domains to be rated as high quality of evidence; otherwise as unclear or low quality. Applicability was categorized as a high, unclear, or low concern, which suggested the agreement between included studies and review question [17]. Optimism bias may exist if a new model outperforms the J-CTO score in its development study but subsequent comparisons fail to support this superiority [15]. Those comparisons whose authors had participated in the development of new models were also considered to have potential optimism bias.

### Statistical analysis

The performance of the J-CTO score was summarized using meta-analyses, separately for 30-min wire crossing and technical success. A logit transformation was used to improve the validity of the extracted C-statistics. The standard error of logit C-statistics was estimated using additional data when no variance was reported [12]. To alleviate the impact from heterogeneity, we adopted restricted maximum likelihood estimation and Hartung–Knapp–Sidik–Jonkman method under a random-effects model [12,18,19]. The heterogeneity across studies was indicated by the Higgins I^2^ statistics, with a value of >75% indicating substantial heterogeneity [20]. Meta-regression analyses were undertaken to explore potential sources of heterogeneity, stratified by recruitment year, mean J-CTO, standard deviation of patient age, and the proportion of retrograde approach. Subgroup analyses were performed to ascertain the effect among different geographic regions and study designs. A sensitivity analysis was conducted to determine the possible influence on pooled estimates from omitting any study. Finally, we examined whether there was any publication bias through Egger’s test [20]. The meta-analysis was performed with Stata, version 15 (StataCorp, TX, USA) using the *metan* and *metareg* command. A two-tailed p-value of <0.05 was considered as statistical significance.

## Results

**Figure 2.**
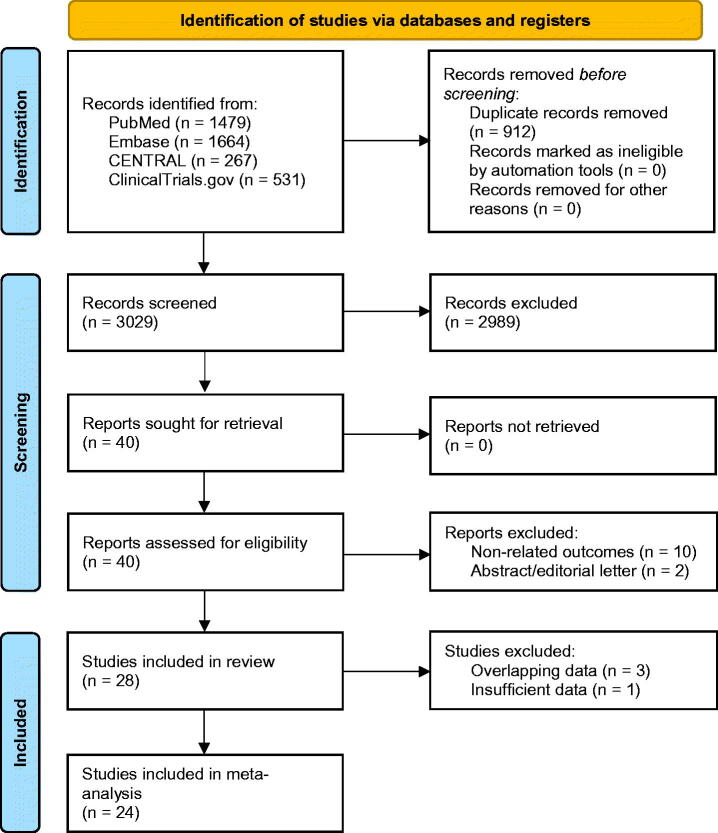
The PRISMA 2020 flow diagram for search and selection.

Of the 3,941 published citations that were captured, 3,029 were further filtered based on their titles and abstracts. After determining the eligibility of 40 full-text articles, we excluded 12 of them due to non-related outcomes (*n* = 10) or article types (*n* = 2) (Table S1). Finally, 28 studies [6–10,21–43] (34,944 lesions) were included in this systematic review, four of which were unavailable for subsequent meta-analyses due to overlapping or insufficient data [7,28,38,39]. The process of search and selection is presented in Figure 2.

### Study characteristics

The main characteristics of included studies are shown in Table 2. Of all 28 studies, three considered successful guidewire crossing within 30 min, 20 considered a technical success, and five did both. Sixteen studies (57%) were prospective and 11 studies (39%) were conducted in multiple centers. Most reports originated from Europe (39%), East Asia (39%), and North America (18%). The median of included lesions was 484 (range: 131–20,627). The mean or median age of participants varied from 54 to 69 years. The event rate of 30-min wire crossing and technical success ranged from 29% to 61% and 60% to 93%, respectively. The definitions of technical success across studies are listed in Table S2.

**Table 2.** Main characteristics of included studies.

Study, year	Time interval	Source of data	Region	Model	Sample size*	Events, %	Age, years	Male, %	Retrograde approach, %
***30-min wire crossing***									
Morino, 2011 [6]	2006-2007	The multi-center CTO registry of Japan	Japan	J-CTO	465 (494)	48.2	26.7%†	86.7	26.9
Li, 2015 [21]	2011-2014	A retrospective, single-center cohort	China	J-CTO (ICA and CTA)	159 (171)	39	65.6 ± 11.9	74.9	NR
Opolski, 2015 [22]	2007-2013	The CT-RECTOR registry	Europe	J-CTO, CT-RECTOR	229 (240)	55	63 ± 10	79.0	11.3
***Technical success***									
Alessandrino, 2015 [26]	2004-2013	A prospective, single-center cohort	France	J-CTO, CL	1657 (1657)	73	64 ± 11	84.5	9.3
Christopoulos, 2015 [7]	2011-2014	The PROGRESS CTO registry	USA	J-CTO	650 (657)	93	65 ± 10	87	29
Nagashima, 2015 [27]	2005-2013	A retrospective, single-center cohort	Japan	J-CTO, SYNTAX	304 (304)	83	68.1 ± 10.2	84.5	13.8
Christopoulos, 2016 [28]	2012-2015	The PROGRESS CTO registry	USA	J-CTO, PROGRESS CTO	762 (781)	92.9	66 ± 10	87	NR
Galassi, 2016 [10]	2005-2014	A retrospective, single-center cohort	Italy	J-CTO, ORA	1019 (1073)	91.9	61.1 ± 9.7	91.4	27.2
Karatasakis, 2016 [36]	2012-2016	The PROGRESS CTO registry	USA	J-CTO, PROGRESS CTO, CL	658 (664)	88	66 ± 10	85	41
Castro-Filho, 2017 [29]	2009-2014	A retrospective, single-center cohort	Brazil	J-CTO	174 (174)	81	59.5 (53.0-65.8)	69.0	1.1
Ellis, 2017 [8]	2014-2015	The OPEN CTO registry	USA	J-CTO, PROGRESS CTO, Basic 7-item model	436 (456)	79.4	63 ± 11	79.0	22.5
Jin, 2017 [30]	1999-2015	The B-CTO registry	South Korea	J-CTO, B-CTO	438 (473)	81.1	61 ± 10	76.3	7.3
Maeremans, 2018 [31]	2014-2015	The RECHARGE registry	Europe	J-CTO, PROGRESS CTO, RECHARGE	880 (880)	84	65 ± 11; 67 ± 11	86.5	NR
Szijgyarto, 2019 [32]	2008-2016	The EuroCTO registry	Europe	J-CTO, CASTLE	20627 (20627)	87	16%†; 64.2 ± 10.4	NR	20.2
Kalnins, 2019 [33]	2007-2016	A retrospective, single-center cohort	Latvia	J-CTO, PROGRESS CTO, CL, CASTLE	551 (551)	82	63.5 ± 10.4	80.0	NR
Su, 2019 [35]	2017-2018	A retrospective, single-center cohort	China	J-CTO, PROGRESS CTO, CL, ORA	246 (246)	82	67.4 ± 10.5	70.7	23.6
Kalogeropoulos, 2020 [34]	2012-2018	A prospective, single-center cohort	UK	J-CTO, CASTLE	660 (660)	78	65.8 ± 10.6	83.8	37
Rigueira, 2020 [43]	2015-2018	A prospective, single-center cohort	Portugal	J-CTO, CTo-aBCDE	334 (377)	60	68 ± 11	75	13.3
Salinas, 2021 [37]	2015-2019	The Iberian registry	Spain and Portugal	J-CTO, PROGRESS CTO, CL, CASTLE	1342 (1342)	77.8	65.17 ± 11.11	84.7	6.6
Mohandes, 2021 [38]	2007-2020	A prospective, single-center cohort	Spain	J-CTO	444 (526)	79.5	65.2 ± 10.9	84.6	14.4
Mohandes, 2021 [42]	2007-2021	A prospective, single-center cohort	Spain	J-CTO, E-CTO	457 (540)	80.1	65 ± 10	84.4	15.4
Gong, 2021 [39]	2015-2018	A retrospective multiple-center cohort	China	J-CTO, PROGRESS CTO, IS-CTO	474 (474)	NR	65 ± 10	82.8	NR
Xiao, 2021 [41]	2018-2019	A prospective, single-center cohort	China	J-CTO, PROGRESS CTO, ORA, RECHARGE, Operator-CTO	130 (144)	91.0	62.81 ± 11.27	80	25.7
***Both 30-min wire crossing and technical success***									
Nombela-Franco, 2013 [9]	2010-2012	A prospective, single-center cohort	Canada	J-CTO	209 (209)	44; 90	67 (60-74)	81.8	53.1
Tan, 2017 [23]	2012-2015	A retrospective single-center cohort	China	J-CTO, CT-RECTOR	191 (191)	54.97; 76	61 ± 11	70.7	15.7
Yu, 2017 [24]	2007-2015	A retrospective, multicenter cohort	South Korea	J-CTO, PROGRESS CTO, CT-RECTOR, CL, KCCT	643 (684)	50; 74	62 (54-70)	80.7	13
Fujino, 2018 [25]	2012-2016	A retrospective, single-center cohort	Japan	J-CTO (ICA and CTA)	205 (218)	29; 83	69 (62-75)	82.4	33.0
Li, 2021 [40]	NR	A retrospective, single-center cohort	China	J-CTO (CTA), CT-RECTOR, RECHARGE (CTA), KCCT	124 (131)	61; 72	54 (43-60)	79	11

B-CTO = Busan single-center Chronic Total Occlusion registry; CASTLE = coronary artery bypass grafting history, ≥70 years of age, stump anatomy (blunt or invisible), tortuosity degree (severe or unseen), length of occlusion (≥20 mm), and extent of calcification (severe); CL = clinical and lesion-specific score; CTA = computed tomographic angiography; CT-RECTOR = Computed Tomography Registry of Chronic Total Occlusion Revascularization; ICA = invasive coronary angiography; NR = not reported; KCCT = Korean Multicenter CTO CT Registry; OPEN CTO = Outcomes, Patient Health Status, and Efficiency in Chronic Total Occlusion; ORA = ostial lesion, Rentrop grade <2, age ≥75 years; PROGRESS-CTO = Prospective Global Registry for the Study of Chronic Total Occlusion Intervention; RECHARGE = REgistry of CrossBoss and Hybrid procedures in FrAnce, the NetheRlands, BelGium and UnitEd Kingdom; SYNTAX = SYNergy between percutaneous coronary intervention with TAXus and cardiac surgery; UK = United Kingdom; USA = United States of America.

Values are expressed as mean ± standard deviation or median (25^th^ percentile-75^th^ percentile) unless otherwise specified.

*Sample size is expressed as the number of patients (lesions) to validate the J-CTO score.

†Proportion of age ≥75 years.

### Features of CTO scores

**Figure 3.**
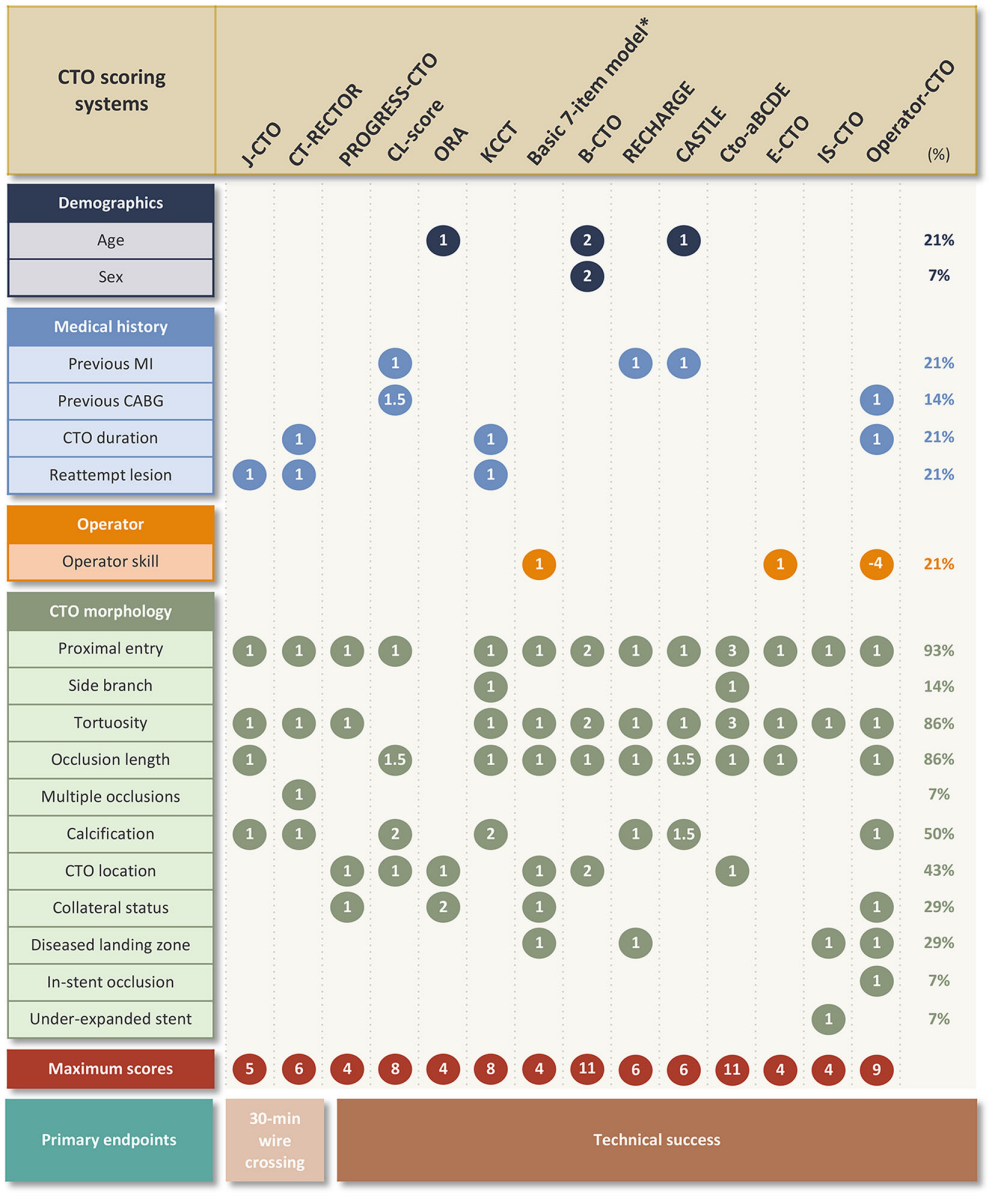
Comparison of variables used in each CTO score. The number in the ball represents the score of each variable. The rightmost column represents the proportion of variables used in CTO scores. *The basic 7-item model is not a traditional risk score but the form of decision tree, so the total score is not the sum of each component.

In total, 14 CTO scores were included in the analysis as well as the SYNTAX score [27]. The PROGRESS CTO score (36%) and the CL-score (21%) were the two comparators of the J-CTO score that were the most commonly reported. The variables included in CTO scores varied widely, including demographic characteristics, medical history, operator skills, and CTO morphology (Figure 3). The most commonly used variables were proximal entry shape (93%), tortuosity (86%), occlusion length (86%), and calcification (50%), all of which are related to lesion morphology. The number of included variables ranged from three to twelve, with a median of six. Most scores were developed for invasive coronary angiography whereas the CT-RECTOR and KCCT scores for computed tomographic angiography (CTA) [22,24].

### Discrimination

**Figure 4.**
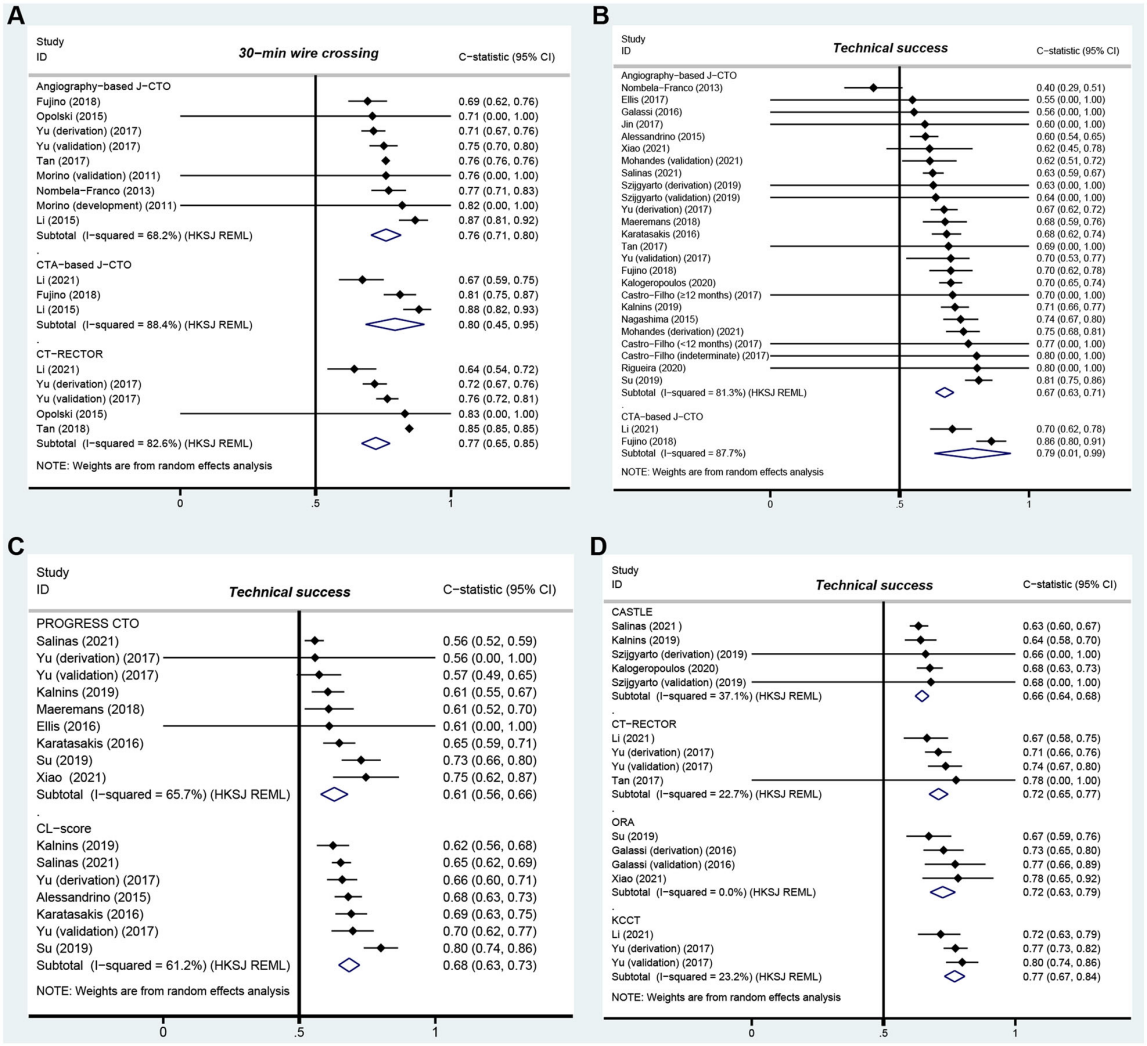
Forest plots of the CTO scores with C-statistics. (A): Predictive performance for 30-min wire crossing; (B–D): Predictive performance for technical success. *95% CI or standard error was not reported. Variance of C-statistics on logit transformation was calculated and used for the meta-analysis. CI: confidence interval; HKSJ: Hartung-Knapp-Sidik-Jonkman; REML: restricted maximum likelihood.

Eight studies reporting data on 2,207 lesions were included to estimate the discrimination for 30-min wire crossing (Figure 4). The synthesized results showed that the angiography-based J-CTO score might be useful in predicting time-efficient wire crossing despite moderate heterogeneity (pooled C-statistic = 0.76, 95% CI 0.71 to 0.80; I^2^ = 68.2%). Geographical region and study design had no significant impact on heterogeneity (Figure S1). A meta-regression analysis was not performed due to the limited number of studies (*n* < 10). In addition, a similar power was found for the CTA-based J-CTO score (pooled C-statistic = 0.80, 95% CI 0.45 to 0.95; I^2^=88.4%) in three studies (1,246 lesions).

Sixteen studies with 29,393 lesions were included in the meta-analysis for technical success. The synthesized results showed that the angiography-based J-CTO score was moderately effective in predicting the ultimate angiographic success accompanied by high heterogeneity (pooled C-statistic = 0.67, 95% CI 0.63 to 0.71; I^2^ = 81.3%). If roughly analysed, the discrimination appeared to be relatively weaker in studies with prospective design or participants from North America (Figure S2). Meta-regression analyses found no contributing factor for heterogeneity: recruitment year (*p* = 0.258), mean J-CTO (*p* = 0.779), standard deviation of age (*p* = 0.528), and retrograde approach (*p* = 0.172). The pooled estimates were not dramatically altered by the removal of any study (Figure S3). No publication bias was revealed through Egger’s test (Figure S4).

Other CTO scores exhibited a wide variety of discrimination (Figure 4). For 30-min wire crossing, the CT-RECTOR score had a strong distinguishing ability (pooled C-statistic = 0.77, 95% CI 0.65 to 0.85), but its significant heterogeneity (I^2^ = 82.6%) should be noted. For technical success, the pooled C-statistics were 0.61 (95% CI 0.56 to 0.66; I^2^ = 65.7%) for PROGRESS CTO, 0.68 (95% CI 0.63 to 0.73; I^2^ = 61.2%) for CL-score, 0.66 (95% CI 0.64 to 0.68; I^2^ = 37.1%) for CASTLE, 0.72 (95% CI 0.65 to 0.67; I^2^ = 22.7%) for CT-RECTOR, 0.72 (95% CI 0.63 to 0.79; I^2^ = 0.0%) for ORA, and 0.77 (95% CI 0.67 to 0.84; I^2^ = 23.2%) for KCCT. There were 58 pairwise comparisons on discrimination, but a statistical test was only available for 19 (33%) comparisons. For 30-min wire crossing, an excess of 5% occurred in seven (54%) comparisons, four of which were statistically significant. For technical success, a relative difference exceeding >5% occurred in 32 (71%) comparisons, seven of which reported a P-value of <0.05. However, some of these differences were inconsistent, suggesting the existence of bias. The metrics of model performance across included studies is shown in Table S3.

### Calibration and risk reclassification

The calibration of the J-CTO score was manifested as Hosmer–Lemeshow statistics in eight studies, four of which were also available for the total O:E ratio. These data suggested good calibration with the J-CTO score except for one study with a Hosmer–Lemeshow *p*-value of 0.001 [34]. A meta-analysis was unavailable for calibration because of its limited data. Nine competing models exhibited good calibration indicated by Hosmer–Lemeshow statistics. There was only one comparison based on O:E ratio: the CASTLE score was better than the J-CTO score, especially in complex cases [34]. Four and three comparisons described net reclassification improvement and integrated discrimination improvement, respectively. There was one independent comparison: the CT-RECTOR score had significant net reclassification improvement for both 30-min wire crossing (30.21%; *p* = 0.027) and technical success (28.94%; *p* = 0.019) compared with the J-CTO score [23] (Table S3).

### Optimism bias

Only five of the competing models exhibited statistical significance when compared to the J-CTO score, despite practically all of them having higher C-statistics with >5% relative difference in their original studies (Table 3). Two articles had some authors involved in the development studies and eight competing models were compared independently with the J-CTO score. Of 33 independent comparisons, the J-CTO score performed worse in eight cases but better in another 13. The PROGRESS CTO score was demonstrated to be less accurate than the J-CTO score, not only by its original study but also by subsequent validations. The superiority of CT-RECTOR over J-CTO was relatively reliable while other models had conflicting results.

**Table 3.** Potential optimism bias in comparing the J-CTO and other competing scores.

Competing scores	Potential optimism bias			Independent comparisons		
	Development study	Higher C-statistic than the J-CTO score (relative differenc*e* > 5%) in the development study?	Involving some authors in the development study	Total number	Relative difference in C-statisti*c* > 5% (Competing model vs. J-CTO)	Statistical significance (Competing model vs. J-CTO)
CT-RECTOR	Opolski, 2015 [22]	Yes*	—	6	4 vs. 0	2 vs. 0
CL	Alessandrino, 2015 [26]	Yes	—	8	0 vs. 1	NS (*n* = 2) [35, 36]
PROGRESS CTO	Christopoulos, 2016 [28]	No	PROGRESS CTO < J-CTO* [36]	11	2 vs. 9	NS (*n* = 1) [35]
ORA	Galassi, 2016 [10]	Yes	—	2	1 vs. 1	0 vs. 1
Basic 7-item model	Ellis, 2017 [8]	Yes*	—	—	—	—
B-CTO	Jin, 2017 [30]	Yes*	—	—	—	—
KCCT	Yu, 2017 [24]	Yes*	—	1	—	—
RECHARGE	Maeremans, 2018 [31]	Yes	—	2	1 vs. 0	NS (*n* = 1) [40]
CASTLE	Szijgyarto, 2019 [32]	Yes	NS^34^	2	0 vs. 1	NS (*n* = 1) [37]
CTo-aBCDE	Rigueira, 2020 [43]	No	—	—	—	—
E-CTO	Mohandes, 2021 [42]	Yes	—	—	—	—
IS-CTO	Gong, 2021 [39]	Yes*	—	—	—	—
Operator-CTO	Xiao, 2021 [41]	Yes	—	—	—	—
SYNTAX	—	—	—	1	0 vs. 1	—

NS = no significance.

*p < 0.05 versus the J-CTO score.

### Methodological evaluation

Overall, we found that all studies had methodological flaws that were stemmed largely from the domain of statistical analysis (Table S4). The absence of a calibration plot or table was primarily responsible for the risk of bias. Conversely, a low proportion of studies were found to have a high risk of bias in the domains of participants (14%), predictors (0%), and outcome (0%). There was unclear concern regarding applicability in nine (32%) studies due to a narrow selection of participants, such as the exclusion of individuals who did not undergo coronary CTA.

## Discussion

This systematic review identified 28 different studies validating the J-CTO score for assessing the difficulty of CTO PCI, 24 of which were considered for the meta-analysis. We also evaluated 14 competing models with 58 head-to-head comparisons to determine their relative performance with the J-CTO score. The main findings can be summarized as follows: 1) the predictive ability of the J-CTO score is strong for efficient guidewire crossing and modest for technical success; 2) there is insufficient evidence to reveal the difference in performance of various CTO scores; and 3) validation studies seldom evaluated the calibration appropriately, leading to methodological shortcomings and potential risk of bias.

Management of CTO is often challenging, even with advanced techniques and dedicated devices. Therefore, the J-CTO score was introduced to grade CTO PCI difficulty by combining five independent predictors [6]. With CTO-PCI techniques continuously evolving, it seems to have become outdated and various updated scores were developed. However, we showed strong discrimination of the J-CTO score in predicting 30-min wire crossing and moderate ability in distinguishing those lesions that are more likely to be recanalized. The heterogeneity was significant but a better performing mathematical model was used to alleviate its impact [18]. Even though meta-regression analysis did not reveal any significant interactions, it may be underpowered to detect such statistical differences due to missing data and a relatively small number of studies.

There is a growing tendency to establish new models but their superiority against the standard one should be interpreted cautiously. For example, the Framingham score was often reported to be inferior to its comparators but inconsistent results were observed in subsequent studies, indicating that such comparison might have been biased by subjective factors [15]. Interestingly, we identified a similar pattern for the J-CTO score. All competing models had exhibited better performance than the J-CTO score in their development studies, except for the PROGRESS CTO score [28]. Nevertheless, conflicting findings were featured afterward, suggesting the existence of potential optimism bias [44]. It is noteworthy that the J-CTO score was developed for 30-min wire crossing whereas most comparisons were based on technical success. This selection of outcome, along with the optimism bias described above, might undermine the credibility of the results [45]. Another concern may be the lack of a formal comparison. Only 33% of the comparisons performed a statistical test. Most validations relied heavily on estimates of discrimination rather than an overall model measurement, which seems to be a common issue among predictive studies that impairs the objectivity of model comparison [46]. Therefore, it may be premature to support the superiority of new scoring systems over the conventional one before there is sufficient evidence.

Admittedly, there remains a degree of uncertainty to the J-CTO score. For instance, a previously failed attempt may be relatively subjective, depending on personal experience. The retrograde approach might be also an influential factor since collateral circulation was not taken into account in the score. Furthermore, there was a relatively low proportion of the retrograde approach in the original study. While improving the success rate, advanced techniques may result in a decrease in model accuracy. However, the validity of the J-CTO score has been confirmed in independent cohorts with a hybrid algorithm [9,36]. Given the complexity of CTO lesions, time efficiency is important for high-volume CTO programs to arrange a reasonable schedule and improve lesion selection. In the present study, the J-CTO score showed moderate predictive ability for procedural success but strong discrimination for 30-min wire crossing, and thus can be used as the cornerstone for assessing CTO lesions at least for now. More accurate scores will be required to predict procedural success, which is far more important than guidewire crossing.

The foundation of interventional therapy for CTO lesions is the careful and repeated interpretation of coronary angiography. Prior to CTO PCI, multiangle bilateral coronary angiography is necessary for the majority of CTO lesions [4]. In this reading process, multiple scores should be combined to maximize their predictive ability and optimize strategy planning. This is not only because of different populations and lesion morphology, but also heterogeneous strengths of interventionalists. Despite sharing some variables, each scoring system has its unique features. The operator could obtain a variety of information based on different scores to make the optimal decision-making. Compared with the non-selective population in the Japanese CTO registry, CL and PROGRESS CTO scores may be more ideal for those operators prone to antegrade and hybrid approaches, respectively [26,28]. Interestingly, we found better discrimination of the CTA-derived J-CTO and CT-RECTOR scores, indicating the incremental value of coronary CTA in quantifying coronary calcium and identifying distal segments [23–25,47]. Although operator skills and experience are critical to the success of CTO interventions, these scores were a powerful assistant for clinical evaluation and can guide intervention strategies, especially when considering the antegrade wire escalation or deciding to initiate a retrograde approach immediately. Additionally, predictive scores may be utilized to identify patients with CTO lesions who are suitable for PCI to ensure a better cardiovascular outcome [5]. Thus, this application should be promoted and become a key part of the CTO algorithm.

Our study has certain limitations. First, some of the included studies were retrospective, which might be inherently influenced by confounding factors. Second, the heterogeneity was significant. The results should be interpreted cautiously even though a random-effects model with adjustment methods was used. Although most validation studies are rated as high risk, this mainly arise from the incompleteness of model evaluation. The comparison among different scores was limited to discrimination. Future efforts are thus required to assess model performance adhering to methodological guidelines.

## Conclusion

This meta-analysis supports the value of the J-CTO score in determining the degree of CTO PCI difficulty, even in the contemporary era of a hybrid algorithm. The discrimination of the J-CTO score is useful for efficient guidewire crossing and moderate for technical success. Current evidence is insufficient to reveal the difference in performance between the J-CTO score and other competing scores. Further high-quality studies evaluating clinical benefits are warranted to mitigate this knowledge gap.

## Author contributions

(I) Conception and design: W Zuo, G Ma; (II) Administrative support: J Lin, G Ma; (III) Provision of study materials or patients: W Zuo, J Lin, R Sun, Y Su,; (IV) Collection and assembly of data: W Zuo, J Lin, R Sun, Y Su; (V) Data analysis and interpretation: W Zuo, J Lin, R Sun, Y Su; (VI) Manuscript writing: All authors; (VII) Final approval of manuscript: All authors.

## Supplementary Material

Supplemental Material

## Data Availability

The data that support the findings of this study are available from the corresponding author, [G Ma], upon reasonable request.
