# Supplementary material for: Performance of the J-CTO score versus other risk scores for predicting procedural difficulty in coronary chronic total occlusion interventions
Source: Ann Med. 2022 Nov 2;54(1):3117–28. doi: 10.1080/07853890.2022.2141466 (PMC9635461; doi:10.1080/07853890.2022.2141466)
Supplement: Supplemental Material [file IANN_A_2141466_SM0244.pdf]

Supplementary Materials

Table of Contents

**Section**	**Page #**
Supplementary file: Search strategy across electronic databases	2
Table S1. List of rejected articles at full-text stage	4
Table S2. Definitions of technical success across included studies	5
Table S3. Metrics of model performance across included studies	7
Table S4. Risk of bias and applicability assessment of the validation studies	10
Figure S1. Subgroup analyses of the angiography-based J-CTO score to predict 30-min wire crossing	12
Figure S2. Subgroup analyses of the angiography-based J-CTO score to predict technical success	13
Figure S3. Sensitivity analyses of pooled logit C-statistics	14
Figure S4. Publication bias of logit C-statistics assessed by Egger's test	15

Supplementary file: Search strategy across electronic databases.

Search terms – PubMed (n=1479)

Search	Query
1	"Coronary Occlusion"[Mesh]
2	Chronic total occlusion[Title/Abstract] OR CTO[Title/Abstract]
3	J-CTO[Title/Abstract]
4	1 OR 2 OR 3
10	"Risk Factors"[Mesh]
11	"Models, Statistical"[Mesh] OR Model*[Title/Abstract]
12	Risk score*[Title/Abstract] OR Score*[Title/Abstract]
13	Clinical tool*[Title/Abstract]
14	Risk prediction model*[Title/Abstract] OR Prediction model*[Title/Abstract] OR Risk prediction score*[Title/Abstract] OR Prediction rule*[Title/Abstract]
15	Risk analysis[Title/Abstract] OR Risk prediction*[Title/Abstract]
16	"Decision Support Techniques"[Mesh] OR Decision support technique*[Title/Abstract] OR Decision support*[Title/Abstract] OR Decision support system*[Title/Abstract]
17	"Risk Management"[Mesh] OR Risk management*[Title/Abstract]
18	"Risk Assessment"[Mesh] OR Risk assessment*[Title/Abstract]
19	10 OR 11 OR 12 OR 13 OR 14 OR 15 OR 16 OR 17 OR 18
20	4 AND 19 Filters: from 2011/1/1 – 2021/12/23

Search terms – Embase (n=1664)

Search	Query
1	‘coronary artery occlusion’/exp OR ‘chronic total occlusion’:ab,ti OR ‘CTO’:ab,ti OR ‘J-CTO’:ab,ti
2	‘statistical model’/exp OR ‘model*’:ab,ti
3	‘risk prediction*’:ab,ti OR ‘risk prediction model*’:ab,ti OR ‘risk prediction score*’:ab,ti OR ‘prediction rule*’:ab,ti
4	‘clinical tool*’:ab,ti
5	‘risk analysis’:ab,ti
6	‘Decision support system’/exp OR ‘decision support system*’
7	‘decision support technique*’:ab,ti OR ‘decision support’:ab,ti
8	‘Risk factor’/exp OR ‘risk factor*’:ab,ti
9	‘Risk management’/exp OR ‘risk management*’:ab,ti
10	‘Risk assessment’/exp OR ‘risk assessment*’:ab,ti
11	‘Prediction model’/exp OR ‘prediction model*’:ab,ti
12	‘Risk score’/exp OR ‘risk score*’:ab,ti OR ‘Score*’:ab,ti
13	2 OR 3 OR 4 OR 5 OR 6 OR 7 OR 8 OR 9 OR 10 OR 11 OR 12
14	1 AND 13
15	14 AND ('article'/it OR 'article in press'/it) Filters: from 2011/1/1 – 2021/12/23

Search terms – CENTRAL (n=267)

Search	Query
#1	MeSH descriptor: [Coronary Occlusion] explode all trees
#2	(chronic total occlusion):ti,ab,kw OR (CTO):ti,ab,kw
#3	(J-CTO):ti,ab,kw
#4	#1 OR #2 OR #3
#5	MeSH descriptor: [Risk Factors] explode all trees
#6	MeSH descriptor: [Models, Statistical] explode all trees
#7	(model*):ti,ab,kw
#8	(risk prediction score*):ti,ab,kw
#9	(clinical tool*):ti,ab,kw
#10	(risk prediction model*):ti,ab,kw OR (prediction rule*):ti,ab,kw OR (prediction model*):ti,ab,kw OR (risk score*):ti,ab,kw
#11	(risk analysis):ti,ab,kw
#12	(risk prediction*):ti,ab,kw
#13	(score*):ti,ab,kw
#14	MeSH descriptor: [Decision Support Techniques] explode all trees
#15	(decision support technique*):ti,ab,kw
#16	(decision support*):ti,ab,kw
#17	(decision support system*):ti,ab,kw
#18	MeSH descriptor: [Risk Management] explode all trees
#19	(risk management*):ti,ab,kw
#20	MeSH descriptor: [Risk Assessment] explode all trees
#21	(risk assessment*):ti,ab,kw
#22	#5 OR #6 OR #7 OR #8 OR #9 OR #10 OR #11 OR #12 OR #13 OR #14 OR #15 OR #16 OR #17 OR #18 OR #19 OR #20 OR #21
#23	#4 AND #22 Filters: from 2011/1/1 – 2021/12/23

Search terms – ClinicalTrials.gov (n=531)

Search	Query
#1	J-CTO
#2	CTO
#3	Chronic total occlusion
#4	#1 OR #2 OR #3 Filters: from inception – 2021/12/23

Table S1. List of rejected articles at full-text stage.

	Author	Year	Title	Reason
1	Maeremans et al	2013	Impact of negative lesion characteristics of chronic total occlusions on procedural outcome and strategy	Non-related outcomes
2	Syrseloudis et al	2013	Increase in J-CTO lesion complexity score explains the disparity between recanalisation success and evolution of chronic total occlusion strategies: insights from a single-centre 10-year experience	Non-related outcomes
3	Chen et al	2015	Predicting successful percutaneous coronary intervention in patients with chronic total occlusion: the incremental value of a novel morphological parameter assessed by computed tomography	Non-related outcomes
4	Boukhris et al	2016	Percutaneous coronary revascularisation for CTO: a novel predictive score of technical failure	Conference abstract
5	Tanaka et al	2016	Impact of J-CTO score on procedural outcome and target lesion revascularisation after percutaneous coronary intervention for chronic total occlusion: a substudy of the J-CTO Registry (Multicentre CTO Registry in Japan)	Non-related outcomes
6	Guelker et al	2017	Validity of the J-CTO Score and the CL-Score for predicting successful CTO recanalization	Non-related outcomes
7	Lembo et al	2017	Predictive Scores of Success in CTO PCI: There Is No Substitute for Operator Experience and Skill	Editorial letter
8	Namazi et al	2017	A Novel Risk Score in Predicting Failure or Success for Antegrade Approach to Percutaneous Coronary Intervention of Chronic Total Occlusion: Antegrade CTO Score	Non-related outcomes
9	Forouzandeh et al	2018	Performance of J-CTO and PROGRESS CTO Scores in Predicting Angiographic Success and Long-term Outcomes of Percutaneous Coronary Interventions for Chronic Total Occlusions	Non-related outcomes
10	Abe et al	2019	Association between J-CTO score and long-term target lesion revascularization rate after successful chronic total coronary occlusion angioplasty (from the J-CTO Registry)	Non-related outcomes
11	Ebisawa et al	2020	Derivation and validation of the J-CTO extension score for pre-procedural prediction of major adverse cardiac and cerebrovascular events in patients with chronic total occlusions	Non-related outcomes
12	Gong et al	2021	The impact of J-CTO score on in-stent chronic total occlusion percutaneous coronary intervention	Non-related outcomes

Table S2. Definitions of technical success across included studies.

Study, year	Definition of technical success
Nombela-Franco, 2013	A restoration of TIMI flow grade 3 and residual stenosis <30% in the occluded artery
Alessandrino, 2015	The achievement of <30% residual diameter stenosis as assessed by quantitative coronary angiography and associated with TIMI flow grade 3
Christopoulos, 2015	Successful CTO revascularization with achievement of <30% residual diameter stenosis within the treated segment and restoration of antegrade TIMI grade 3 flow
Nagashima, 2015	Successful balloon dilatation of the lesion with or without stent placement and less than 40 % residual stenosis
Christopoulos, 2016	Successful CTO revascularization with achievement of <30% residual diameter stenosis within the treated segment and restoration of antegrade TIMI flow grade 3
Galassi, 2016	Final residual stenosis <20% by visual estimation and TIMI flow grade 3 after CTO recanalization
Karatasakis, 2016	Successful CTO revascularization with achievement of <30% residual diameter stenosis within the treated segment and restoration of TIMI grade 3 antegrade flow
Castro-Filho, 2017	Restoration of TIMI grade 3 and residual stenosis <30% in the occluded artery
Ellis, 2017	Restoration of TIMI flow grade 3 into branches constituting at least one-half of the segments distal to the occlusion and no stenosis >50% within the site of occlusion
Jin, 2017	<20% residual stenosis with TIMI flow grade ≥2 by visual estimation of the angiograms
Tan, 2017	Successful CTO revascularization with achievement of <30% residual diameter stenosis within the treated segment at any time and restoration of antegrade TIMI grade 3 flow
Yu, 2017	Diameter stenosis <50% and restoration of TIMI grade 3 flow
Maeremans, 2018	Successful CTO revascularization with achievement of <30% residual diameter stenosis within the stented segment and restoration of TIMI grade 3 antegrade flow
Fujino, 2018	Post-PCI TIMI flow grade 3 and residual stenosis <30%
Szijgyarto, 2019	Residual stenosis of <10% at the end of the procedure with TIMI flow grade 3 antegrade flow
Kalnins, 2019	Complete restoration of the antegrade blood flow (TIMI flow grade 3) with an arterial lumen diameter reduction to less than 10% in the culprit CTO vessel
Su, 2019	Complete restoration of the antegrade blood flow (TIMI flow grade 3 with an arterial lumen diameter reduction to less than 30% in the culprit CTO vessel)
Kalogeropoulos, 2020	Successful CTO revascularization with achievement of <30% residual diameter stenosis within the treated segment and restoration of TIMI grade 3 antegrade flow
Rigueira, 2020	PCI with stent placement resulting in TIMI flow 3

Salinas, 2021	CTO recanalization with final TIMI 3 flow
Mohandes, 2021	Successful recanalization of the occluded artery with final TIMI flow grade III and residual lesion <30%
Mohandes, 2021	Achievement of TIMI grade 2 or greater antegrade flow in all ≥ 2.5 mm distal branches with < 30% residual stenosis of the target CTO lesion
Gong, 2021	Residual stenosis of < 30% and restoration of antegrade TIMI flow grade 3 in the CTO segment
Li, 2021	Successful CTO revascularization with the achievement of < 20% residual diameter stenosis within the stented segment and the restoration of a TIMI = 3
Xiao, 2021	The restoration of the forward blood flow TIMI3, the achievement of <30% stenosis in the stent segment, and no related complications during the operation

Abbreviations: TIMI: thrombolysis in myocardial infarction; CTO: chronic total occlusion; PCI: percutaneous coronary intervention.

Table S3. Metrics of model performance across included studies.

Study, year	Score	C-statistic (95% CI) Derivation cohort	C-statistic (95% CI) Validation cohort	Calibration H-L *P* value	Calibration O:E ratio (95% CI)
30-min wire crossing					
Morino, 2011	J-CTO (ICA)	0.82	0.76	—	—
Nombela-Franco, 2013	J-CTO (ICA)	—	0.770 (0.706-0.834)	0.464	1.00 (0.80-1.20)
Li, 2015	J-CTO (ICA)	—	0.868 (0.808-0.915)	—	—
	J-CTO (CTA)	—	0.882 (0.824-0.927)[NS]	—	—
Opolski, 2015	J-CTO (ICA)	—	0.71	—	—
	CT-RECTOR	—	0.83*	—	—
Tan, 2017	J-CTO (ICA)	—	0.7592	—	—
	CT-RECTOR	—	0.8462*	—	—
Yu, 2017	J-CTO (ICA)	0.714 (0.669-0.758)	0.752 (0.699-0.803)	—	—
	CT-RECTOR	0.718 (0.674-0.763)	0.765 (0.724-0.806)	—	—
	PROGRESS CTO	0.651 (0.504-0.700)	0.603 (0.534-0.672)	—	—
	CL	0.682 (0.624-0.730)	0.737 (0.672-0.801)	—	—
	KCCT	0.776 (0.735-0.818)*	0.809 (0.754-0.864)*	—	—
Fujino, 2018	J-CTO (ICA)	—	0.692 (0.621-0.764)	—	—
	J-CTO (CTA)	—	0.812 (0.752-0.871)*	—	—
Li, 2021	J-CTO (CTA)	—	0.673 (0.586-0.752)	—	—
	CT-RECTOR	—	0.643 (0.544-0.724)	—	—
	RECHARGE (CTA)	—	0.708 (0.622-0.784)[NS]	—	—
	KCCT	—	0.703 (0.617-0.780)	—	—
Technical success					
Nombela-Franco, 2013	J-CTO (ICA)	—	0.399 (0.286-0.511)	—	—
Alessandrino, 2015	J-CTO (ICA)	—	0.60 (0.54-0.65)	—	—
	CL	—	0.68 (0.63-0.73)	—	—
Christopoulos, 2015	J-CTO (ICA)	—	0.705	0.743	—
Nagashima, 2015	J-CTO (ICA)	—	0.736 (0.669-0.804)	—	—
	SYNTAX	—	0.687 (0.606-0.769)	—	—
Christopoulos, 2016	J-CTO (ICA)	—	0.746	—	—
	PROGRESS CTO	0.778	0.720	0.268; 0.770	—
Galassi, 2016	J-CTO (ICA)	0.556		0.05	—
	ORA	0.728 (0.652-0.804)	0.772 (0.657-0.887)	—	—
Karatasakis, 2016	J-CTO (ICA)	—	0.682 (0.625-0.738)	0.117	—
	PROGRESS CTO	—	0.647 (0.588-0.706)[NS]	0.067	—
	CL	—	0.691 (0.633-0.749)[NS]	0.846	—
Castro-Filho, 2017	J-CTO (indeterminate)	—	0.798	0.77	
	J-CTO (<12 months)	—	0.766	0.76	1.01 (0.78-1.25)
	J-CTO (≥12	—	0.705	0.89	

	months)				
Ellis, 2017	J-CTO (ICA)	—	0.55	—	—
	PROGRESS CTO	—	0.61	—	—
	Basic 7-item model	0.753	0.738*	—	—
Jin, 2017	J-CTO (ICA)	—	0.598	—	—
	B-CTO	—	0.681*	0.829	—
Tan, 2017	J-CTO (ICA)	—	0.6894	—	—
	CT-RECTOR	—	0.7754*	—	—
Yu, 2017	J-CTO (ICA)	0.672 (0.620-0.724)	0.698 (0.526-0.770)	—	—
	CT-RECTOR	0.708 (0.658-0.758)	0.736 (0.669-0.798)	—	—
	PROGRESS CTO	0.558	0.573 (0.491-0.654)	—	—
	CL	0.658 (0.602-0.713)	0.697 (0.619-0.774)	—	—
	KCCT	0.773 (0.728-0.819)*	0.799 (0.738-0.860)*	—	—
Maeremans, 2018	J-CTO (ICA)	—	0.676 (0.59-0.76)	—	—
	PROGRESS CTO	—	0.608 (0.52-0.70)	—	—
	RECHARGE	0.783 (0.74-0.83)	0.711 (0.63-0.79)	0.15; 0.83	—
Fujino, 2018	J-CTO (ICA)	—	0.698 (0.615-0.782)	—	—
	J-CTO (CTA)	—	0.855 (0.797-0.912)*	—	—
Szijgyarto, 2019	J-CTO (ICA)	0.63	0.64	—	—
	CASTLE	0.66	0.68	0.1	0.94 (0.90-0.97)
Kalnins, 2019	J-CTO (ICA)	—	0.714 (0.660-0.768)	—	—
	PROGRESS CTO	—	0.605 (0.546-0.665)	—	—
	CL	—	0.624 (0.565-0.683)	—	—
	CASTLE	—	0.641 (0.581-0.701)	—	—
Su, 2019	J-CTO (ICA)	—	0.806 (0.753-0.859)	—	—
	PROGRESS CTO	—	0.727 (0.656-0.799)^{NS}	—	—
	CL	—	0.800 (0.737-0.863)^{NS}	—	—
	ORA	—	0.672 (0.587-0.757)*	—	—
Kalogeropoulos,	J-CTO (ICA)	—	0.698 (0.653-0.742)	0.001	0.98 (0.90-1.07)
2020	CASTLE	—	0.676 (0.627-0.725)^{NS}	0.858	1.01 (0.92-1.10)
Rigueira, 2020	J-CTO (ICA)	—	0.80	—	—
	CTo-aBCDE	0.831		—	—
Salinas, 2021	J-CTO (ICA)	—	0.628 (0.59-0.67)	0.01	—
	PROGRESS CTO	—	0.557 (0.52-0.59)	0.001	—
	CL	—	0.652 (0.62-0.69)	0.263	—
	CASTLE	—	0.633 (0.60-0.67)^{NS}	0.382	—
Mohandes, 2021	J-CTO (ICA)	—	0.696 (0.639-0.752)	0.43	1.00 (0.90-1.10)
Mohandes, 2021	J-CTO (ICA)	0.747 (0.68-0.81)	0.617 (0.51-0.72)	—	—
	E-CTO	0.768 (0.706-0.830)	0.704 (0.613-0.796)	0.87; 0.16	—
Gong, 2021	J-CTO (ICA)	—	0.642	—	—
	PROGRESS CTO	—	0.579	—	—
	IS-CTO	0.973	0.976*	0.072; 0.632	—

Li, 2021	J-CTO (CTA)	—	0.704 (0.618-0.780)	—	—
	CT-RECTOR	—	0.665 (0.577-0.745)	—	—
	RECHARGE (CTA)	—	0.718 (0.633-0.793)[NS]	—	—
	KCCT	—	0.717 (0.631-0.792)	—	—
Xiao, 2021	J-CTO (ICA)	—	0.616 (0.449-0.782)	0.409	—
	PROGRESS CTO	—	0.745 (0.624-0.866)	0.603	—
	ORA	—	0.783 (0.648-0.917)	0.299	—
	RECHARGE	—	0.738 (0.603-0.873)	0.660	—
	Operator-CTO	—	0.901 (0.821-0.982)	0.883	—

*P <0.05 versus the J-CTO score. NS: no significance

Table S4. Risk of bias and applicability assessment of the validation studies.

Study, year	ROB				Applicability			Overall	
	Participants	Predictors	Outcome	Analysis	Participants	Predictors	Outcome	ROB	Applicability
Morino, 2011	+	+	+	−	+	+	+	−	+
Nombela-Franco, 2013	+	+	+	−	+	+	+	−	+
Alessandrino, 2015	−	?	+	−	?	+	+	−	?
Christopoulos, 2015	+	+	+	−	+	+	+	−	+
Nagashima, 2015	−	+	+	−	?	+	+	−	?
Opolski, 2015	+	+	+	−	?	+	+	−	?
Li, 2015	+	+	+	−	?	+	+	−	?
Christopoulos, 2016	+	+	+	−	+	+	+	−	+
Galassi, 2016	+	?	+	−	+	+	+	−	+
Karatasakis, 2016	+	+	+	−	+	+	+	−	+
Castro-Filho, 2017	+	?	+	−	+	+	+	−	+
Ellis, 2017	+	?	+	−	+	+	+	−	+
Jin, 2017	+	+	+	−	+	+	+	−	+
Tan, 2017	+	+	+	−	?	+	+	−	?
Yu, 2017	+	?	+	−	?	+	+	−	?
Maeremans, 2017	+	+	+	−	+	+	+	−	+
Fujino, 2018	+	+	+	−	?	+	+	−	?
Szijgyarto, 2019	+	+	+	−	+	+	+	−	+
Kalnins, 2019	−	?	+	−	?	+	+	−	?
Su, 2019	?	?	+	−	+	+	+	−	+
Kalogeropoulos, 2020	+	+	+	−	+	+	+	−	+
Rigueira, 2020	?	?	+	−	+	+	+	−	+
Salinas, 2021	?	?	+	−	+	+	+	−	+
Mohandes, 2021	?	+	+	−	+	+	+	−	+
Mohandes, 2021	?	+	+	−	+	+	+	−	+
Gong, 2021	−	?	+	−	?	+	+	−	?

Li, 2021	+	+	+	–	+	+	+	–	+
Xiao, 2021	+	+	+	–	+	+	+	–	+

ROB: risk of bias. + indicates low ROB/low concern regarding applicability; - indicates high ROB/high concern regarding applicability; and ? indicates unclear ROB/unclear concern regarding applicability.

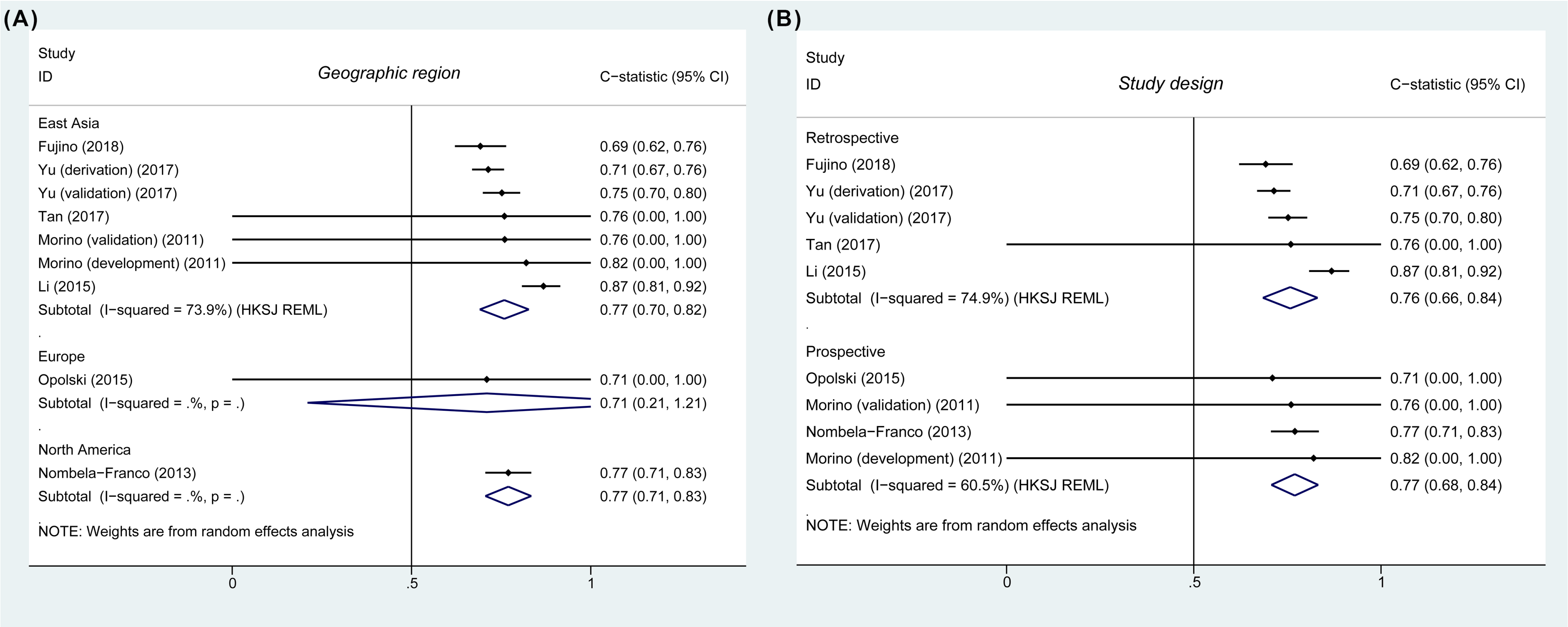

Figure S1. Subgroup analysis of the angiography-based J-CTO score to predict 30-min wire crossing based on geographic region (A) and study design (B). CI: confidence interval.

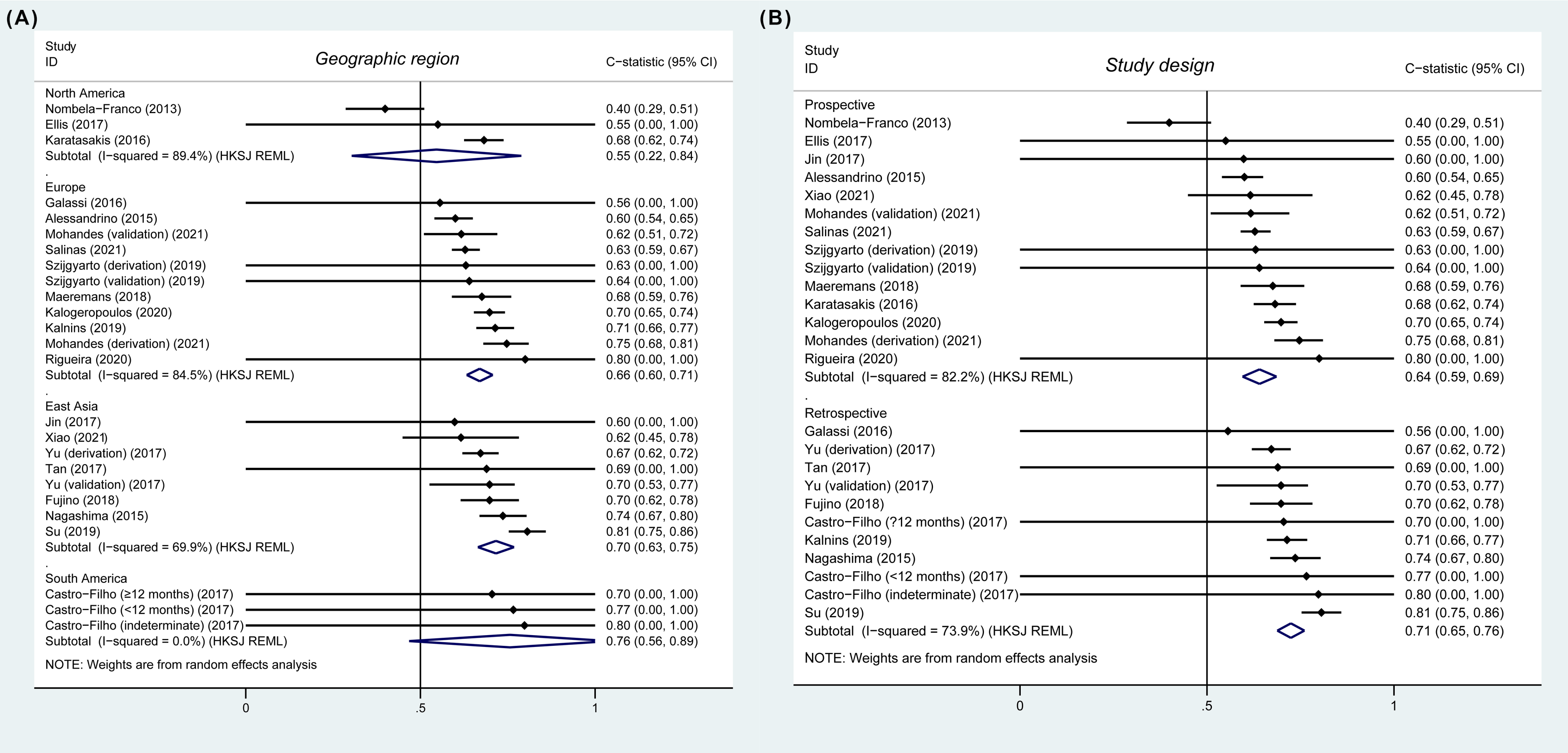

Figure S2. Subgroup analysis of the angiography-based J-CTO score to predict technical success based on geographic region (A) and study design (B). CI: confidence interval.

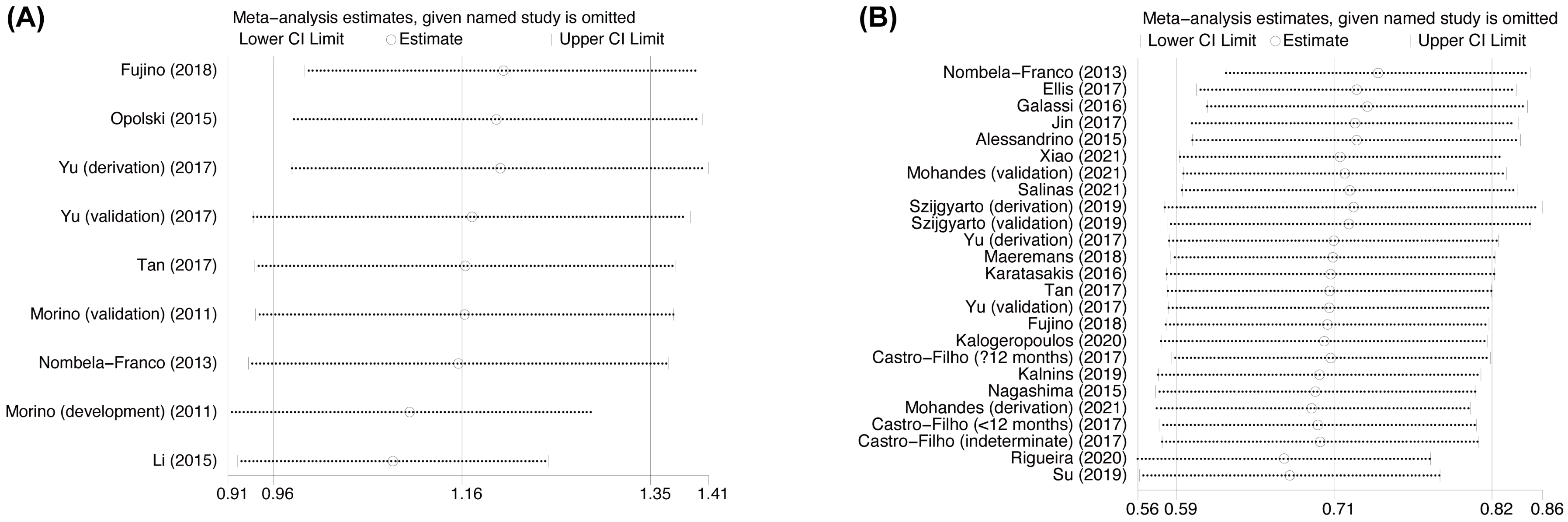

Figure S3. Sensitivity analyses of pooled logit C-statistics to predict 30-min wire crossing (A) and technical success (B). CI: confidence interval.

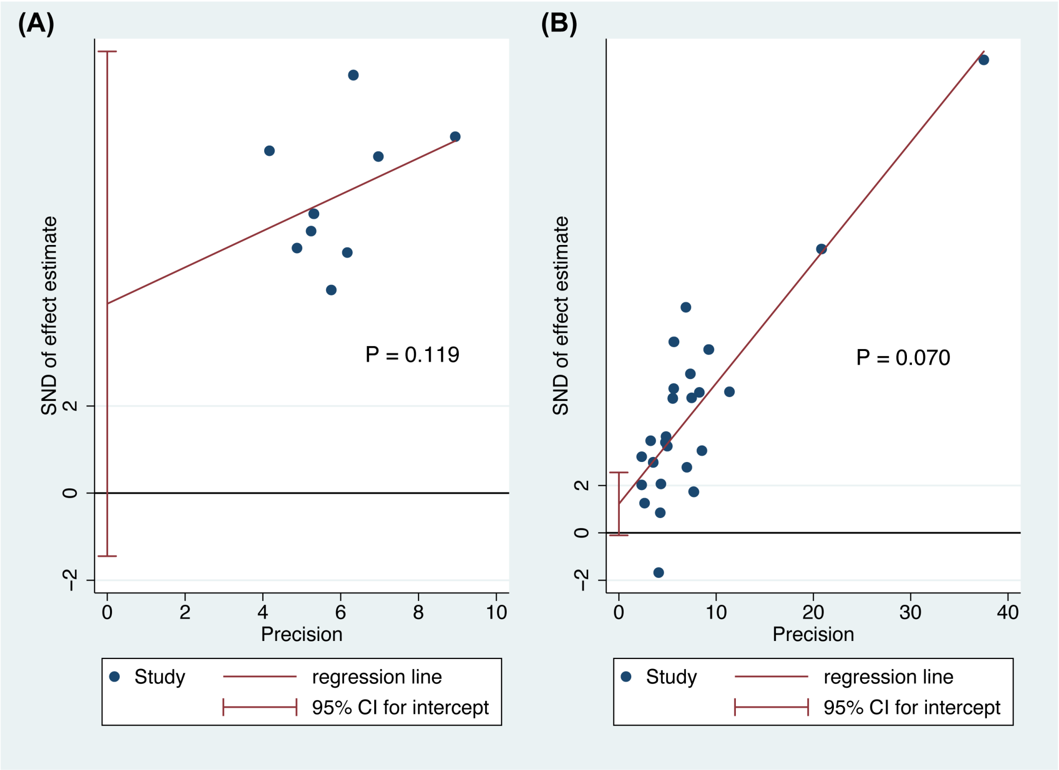

Figure S4. Publication bias of logit C-statistics assessed by Egger's test to predict 30-min wire crossing (A) and technical success (B). SND: standard normal deviate; CI: confidence interval.
